# High-intensity walking in midlife is associated with improved memory in physically capable older adults

**DOI:** 10.1186/s13195-023-01293-8

**Published:** 2023-08-29

**Authors:** Young Min Choe, Guk-Hee Suh, Boung Chul Lee, Ihn-Geun Choi, Hyun Soo Kim, Jong Wan Kim, Jaeuk Hwang, Dahyun Yi, Jee Wook Kim

**Affiliations:** 1 Department of Psychiatry, Hallym University College of Medicine, Chuncheon, Gangwon 24252 Republic of Korea; 2 Department of Neuropsychiatry, Hallym University Dongtan Sacred Heart Hospital, 7 Keunjaebong-Gil, Hwaseong, Gyeonggi 18450 Republic of Korea; 3 Department of Neuropsychiatry, Hallym University Hangang Sacred Heart Hospital, Seoul, 07247 Republic of Korea; 4 Department of Psychiatry, Seoul W Psychiatric Office, Seoul, 08594 Republic of Korea; 5 Department of Laboratory Medicine, Hallym University Dongtan Sacred Heart Hospital, 7 Keunjaebong-Gil, Hwaseong, Gyeonggi 18450 Republic of Korea; 6 Department of Surgery, Hallym University Dongtan Sacred Heart Hospital, 7 Keunjaebong-Gil, Hwaseong, Gyeonggi 18450 Republic of Korea; 7 Department of Psychiatry, Soonchunhyang University Hospital Seoul, Seoul, 04401 Republic of Korea; 8 Institute of Human Behavioral Medicine, Medical Research Center, Seoul National University, Seoul, 03080 Republic of Korea

**Keywords:** Walking, Memory, Alzheimer’s disease, Intensity, Midlife-initiated

## Abstract

**Background:**

Little is known about the associations of midlife- and late life-initiated walking with Alzheimer’s disease (AD)-related cognitive decline in humans. We aimed to investigate whether high-intensity, prolonged, midlife-initiated walking is associated with changes in AD-related cognitive decline in physically capable older adults.

**Methods:**

We studied 188 physically capable participants aged 65–90 years without dementia who underwent comprehensive clinical assessment, including of their walking modality (i.e., intensity, duration, midlife- or late life-onset), memory- or non-memory and total cognitive performance, and blood or nutritional biomarkers.

**Results:**

The walking group showed better episodic memory (*B* = 2.852, *SE* = 1.214, *β* = 0.144, *p* = 0.020), but not non-memory cognition, than the non-walking group. High-intensity walking starting in midlife was significantly associated with better episodic memory (*B* = 9.360, *SE* = 3.314, *β* = 0.446, *p* = 0.005) compared to the non-walking group. In contrast, there were no differences in cognition according to walking duration, regardless of the onset time. The walking group also showed a similar association with overall cognition.

**Conclusions:**

Among physically capable older adults without dementia, walking, particularly at high intensity and starting in midlife, is associated with improved episodic memory, an AD-related cognitive domain. Further attention should be paid to the role of walking in terms of AD prevention.

**Supplementary Information:**

The online version contains supplementary material available at 10.1186/s13195-023-01293-8.

## Background

Alzheimer’s disease (AD) is the common progressive neurodegenerative disease in old adults [1]. As cognitive decline in the disease begins several years before individuals develop dementia, progressive decline in cognitive function is the primary clinical manifestation of AD [2]. A plenty of studies have shown sustained cognitive decline in episodic memory across longitudinal AD trajectories that includes preclinical, prodromal, and early stage of AD, indicating that episodic memory is the first cognitive domain to show a change in AD [3–6].

To prevent or slow down AD and related cognitive decline, an evidence-based strategy focused on lifestyle changes, such as physical activity, is required because of a lack of effective drugs for AD and related cognitive decline. Accumulating evidence suggests that physical activity is a protective factor against AD and related cognitive decline [7–10]. Although several studies have evaluated the effects of global physical activity on AD and related cognitive decline [7, 8], an increasing number of studies have found that even a single physical activity may also have beneficial effect [9, 10].

Walking is one of the single physical activities that may improve AD-related cognitive decline. It is the most accessible physical activity and can be engaged in throughout the life course. Walking can be performed alone or in groups, without any restrictions regarding the time and place, and with no additional costs or learning requirements. A slow walking speed is a major risk factor for the development and exacerbation of AD, suggesting an inverse association between walking and AD-related cognitive decline [11–15]. Therefore, it is essential to investigate the association between walking and AD-related cognitive decline. Several studies have suggested that walking protects against AD and related cognitive decline [16, 17]. A cohort study [16] showed that older women with higher baseline levels of walking were less likely to develop cognitive decline over the 6–8-year follow-up. Another cohort study [17] showed that physically capable elderly men who walked regularly had a reduced risk of AD dementia. However, little is known about the associations of walking intensity, duration, and initiation time (i.e., midlife or late life) with cognitive functions, i.e., episodic memory, non-memory, and overall cognition in humans.

We hypothesized that high-intensity midlife-initiated walking is associated with episodic memory, which is the first cognitive domain to show a change in AD [3–6], in physically capable older adults. Additionally, we investigated the associations of walking with non-memory and overall cognition for comparative purposes.

## Methods

### Participants

This study is part of the General Lifestyle and AD (GLAD) study, an ongoing prospective cohort study that began in 2020. As of September 2022, we enrolled 188 non-demented adults aged 65–90 years, including 107 cognitively normal (CN) adults and 81 with mild cognitive impairment (MCI). Participants were recruited from among individuals who attended a dementia screening program at the memory clinic of Hallym University Dongtan Sacred Heart Hospital, Hwaseong, South Korea. The volunteers were screened for their eligibility for study participation. Additional volunteers were recruited from the community through recommendations by participants, family members, friends, and acquaintances. The CN group consisted of participants with a Clinical Dementia Rating [18] score of 0 and no MCI or dementia. Participants with MCI fulfilled the consensus criteria for amnestic MCI, including memory complaints confirmed by an informant, objective memory impairment, preservation of global cognitive function, independence in functional activities, and the absence of dementia. Objective memory impairment was indicated by an age-, education-, and sex-adjusted *z*-score <  − 1.0 on at least one of the four episodic memory test components of the Korean version of the Consortium to Establish a Registry for Alzheimer’s Disease (CERAD) neuropsychological battery [19, 20]: word list memory, word list recall, word list recognition, and the constructional recall test [19–21]. On the Clinical Dementia Rating, individuals with a score of 0.5 were classified as MCI. We excluded participants with a major psychiatric illness, significant neurological or medical condition, comorbidity that could affect mental function, illiteracy, visual/hearing difficulties, or severe communication or behavioral problems that would make clinical examinations difficult; we also excluded those currently using an investigational drug.

### Standard protocol approvals, registrations, and participants consent

The study protocol was approved by the Institutional Review Board of Hallym University Dongtan Sacred Heart Hospital and the study was conducted in accordance with the Declaration of Helsinki. All participants provided informed consent.

### Clinical assessments

The participants underwent standardized clinical assessments supervised by trained psychiatrists. The clinical assessment protocol incorporated the CERAD clinical and neuropsychological battery [19, 20] and was administered by trained neuropsychologists [21]. AD-related cognitive function was assessed based on episodic memory, which is the first cognitive domain to show changes in AD [3–6], and compared with non-memory cognition. An episodic memory score was derived by summing the scores of the four episodic memory tests (word list memory, word list recall, word list recognition, and constructional recall) in the CERAD neuropsychological battery. The non-memory score was calculated by summing the scores of the three non-memory tests (verbal fluency, modified Boston naming test, and constructional praxis) in the CERAD neuropsychological battery. We used the Geriatric Depression Scale (GDS) [22, 23] to measure the severity of depressive symptoms and categorize the participants into two groups (normal: GDS score of 0–9; depressed: score ≥ 10) [22]. The participants were categorized into three groups based on their annual income: below the minimum cost of living (MCL), above the MCL but below twice the MCL, and at least twice the MCL (http://www.law.go.kr). The MCL was determined according to administrative data published by the Ministry of Health and Welfare, Republic of Korea in November, 2012. The MCL was 572,168 Korean Won (equivalent to US$ 507.9) per month for a single-person household, which increased by 286,840 Korean Won (equivalent to US$ 254.6) per month for each additional household member.

Alcohol intake status (never, former, or current drinker) was determined based on interviews conducted by trained researchers and a review of the medical records. Vascular risk factors (hypertension, diabetes mellitus, dyslipidemia, coronary heart disease, transient ischemic attack, and stroke) were assessed based on data collected by trained researchers during systematic interviews of the participants and their families. A vascular risk score (VRS) was calculated based on the number of vascular risk factors present and is reported as a percentage [24]. Body mass index (BMI) was calculated as the weight in kilograms divided by the height in meters squared. Based on the BMI, we categorized participants as underweight (< 18.5 kg/m^2^), normal weight (18.5–24.9 kg/m^2^), or overweight/obese (> 24.9 kg/m^2^) according to the World Health Organization guidelines (https://www.who.int/europe/news-room/fact-sheets/item/a-healthy-lifestyle---who-recommendations). The accuracy of the information was ensured by interviewing reliable informants.

### Assessment of walking

The participants were systematically interviewed to identify their lifetime walking activity using the format adopted by the interviewer-administered Lifetime Total Physical Activity Questionnaire, which has sufficient reliability and validity [25, 26]. Walking activities were evaluated based on the following questions: “Have you ever walked for exercise or sports as well as for daily life (e.g., walking to school, work, home or bus stop, etc.)? We only include walking activities that you have done at least 10 times in your life. The minimum for walking activity is 32 h per year, or 40 min per week per year, or 2 h per week for 4 months, if seasonal.”, “How intense was your walking activity?”, and “Did the walking activity increase your heart rate or make you sweat?”. Additionally, the participants were asked to describe the walking activity, i.e., age when it started and ended, frequency, amount per activity, and intensity (light, moderate, or vigorous).

Walking activity was defined as walking for 32 h per year, 40 min per week per year, or 2 h per week for 4 months, if seasonal. Based on this definition, the participants were categorized into non-walking and walking groups. Physical activity guidelines for older adults recommend regular moderate-intensity walking or vigorous-intensity activity to achieve health benefits [27]. The guidelines from the Mayo Clinic were applied to assess the intensity levels: moderate-intensity level (breathing quickens, but not out of breath; a light sweat after about 10 min of activity; can carry on a conversation, but cannot sing) and vigorous-intensity level (breathing is deep and rapid; a sweat after only a few minutes of activity; cannot say more than a few words without pausing for breath) (https://www.mayoclinic.org/healthy-lifestyle/fitness/in-depth/exercise-intensity/art-20046887) Based on these guidelines, our participants were categorized into non-walking (not meeting the minimum level of walking activity), low-intensity walking (less than moderate-intensity walking), and high-intensity walking (moderate-to-vigorous-intensity walking) groups. Previous studies of the effects of walking and physical activity showed differences in the risk of dementia among short-duration (or distance), intermediate-duration (or distance), and long-duration (or distance) groups.^16,34,35^ Based on those findings, our participants were categorized into non-walking (not meeting the minimum level of walking activity), short-duration (walking activity group: walking ≤ 360 min per week), and long-duration (high walking activity group: walking > 360 min per week) groups. We investigated the associations of midlife- and late life-initiated walking with AD-related cognitive decline because of possible variation in the association of walking with AD-related cognitive decline according to the timing of initiation of the walking activity [28, 29]. The participants were categorized into two subgroups according to the time of walking initiation, i.e., midlife-initiated (aged 40–64 years) and late life-initiated (aged ≥ 65 years).

### Assessment of overall physical activity

Physical activities were evaluated using the Korean-version of the Physical Activity Scale for the Elderly (PASE) [30, 31], which has sufficient reliability and validity. Trained researchers assessed the frequency, duration and intensity of participants’ leisure, household, and occupational activities during the previous week. The test items were weighted and a PASE total score was obtained by summing the PASE subscale scores for leisure, household, and occupational activities. A higher score indicated greater physical activity.

### Assessment of motor signs

The participants were interviewed, and a gait score was derived based on the motor subscale of the Unified Parkinson’s Disease Rating Scale (UPDRS) [32] to determine the effect of walking on AD-related cognitive domain. Walking is a complex motor movement that requires coordination of all body parts, as well as gait [33]; it can be affected by pre-existing brain diseases.

### Assessment of dietary patterns

Dietary patterns were also investigated in the interviews by the Mini Nutritional Assessment, including food types consumed (such as proteins, fruits, and vegetables) and nutritional status (e.g., changes in food intake over the past 3 months because of loss of appetite, digestive problems, and chewing and swallowing difficulties [34].

### Blood test and APOE4 genotyping

After an overnight fast, blood samples were obtained by venipuncture in the morning (8–9 a.m.). The hemoglobin level was determined using the XN-3000 automated hematologic analyzer and dedicated reagents (Sysmex, Kobe, Japan). Albumin, glucose, and high-density lipoprotein (HDL) and low-density lipoprotein (LDL) cholesterol were measured using the COBAS c702 analyzer and dedicated reagents (Roche Diagnostics, Mannheim, Germany). APOE genotype was determined using the APOE ACE Genotyping Kit (Seegene, Seoul, Korea). APOE4 positivity was defined as the presence of at least one ε4 allele.

### Statistical analyses

To examine the relationship between walking activity and cognition, multiple linear regression analyses were performed with walking activity as the independent variable and cognition as the dependent variable. For these analyses, we used the “no activity” as the reference. As various factors may influence the association between walking activity and cognition, we identified potential confounders, such as age, sex, APOE4, education, clinical diagnosis, depression, annual income, alcohol intake, vascular risk factors, BMI, dietary patterns (including food and nutrient types such as protein, fruits, and vegetables), blood nutritional markers (hemoglobin, albumin, and glucose), and overall physical activity. We tested two models adjusted for covariates in a stepwise manner. The first model included age, sex, and APOE4 as covariates, while the second model also included education, clinical diagnosis, GDS score, annual income, alcohol intake, VRS, BMI, dietary patterns, blood nutritional markers, and the PASE total score. The assumptions of normality and homoscedasticity of the residuals and collinearity of the variables were tested and verified to check the quality of the regression analyses using normal predicted probability plots, scatter plots, and variance inflation factor values, respectively.

The moderating effect of covariates (age, sex, APOE4, education, clinical diagnosis, GDS score, annual income, VRS, and PASE total score), including two-way interaction terms, on the association between walking and cognition was examined in multiple linear regression analyses. Linear regression analyses were repeated in cases of significant interaction terms. For sensitivity analyses, the analyses were repeated for participants reporting no decrease in food intake over the past 3 months due to loss of appetite, digestive problems, or chewing or swallowing difficulties, to preclude any possible influence of physical or mental conditions on walking activity and cognition. The statistical analyses were performed using the SPSS software (version 27.0; IBM Corp, Armonk, NY, USA).

## Results

### Participant characteristics

Table 1 presents the demographic and clinical characteristics of the participants according to walking status, and Table 2 presents those data according to walking intensity and duration. All participants were physically capable, i.e., able to walk without assistance (UPDRS gait score $\le$ 2). Of the 188 participants, 125 were included in the walking group (high intensity, *n* = 57; low intensity, *n* = 68; high duration, *n* = 50, low duration, *n* = 75) and 66 in the non-walking group.

**Table 1:** Baseline participant characteristics according to walking status

	Overall	Non-walking	Walking	*p*
*n*	188	63	125	
Age, years	72.01 (5.16)	72.67 (5.23)	71.67 (5.11)	0.213^a^
Female, no. (%)	133 (70.74)	45 (71.43)	88 (70.40)	0.884^b^
**Education, no. (%)**				0.197^b^
≥13 years	39 (20.74)	9 (14.29)	30 (24.00)	
10–12 years	50 (26.60)	14 (22.22)	36 (28.80)	
4–9 years	82 (43.62)	33 (52.38)	49 (39.20)	
0–3 years	17 (9.04)	7 (11.11)	10 (8.00)	
MMSE	25.67 (3.42)	24.76 (3.80)	26.13 (3.14)	0.009^a^
APOE4 positivity, no. (%)	37 (19.68)	13 (20.63)	24 (19.20)	0.815^b^
Clinical diagnosis, CN, no. (%)	107 (56.91)	33 (52.38)	74 (59.20)	0.373^b^
**Walking intensity**				< 0.001^b^
None	63 (33.51)	63 (100.00)	0 (0.00)	
Low (less than moderate-intensity)	68 (4.93)	0 (0.00)	68 (54.40)	
High (moderate to vigorous-intensity)	57 (30.32)	0 (0.00)	57 (45.60)	
**Walking duration**				< 0.001^b^
None	63 (33.51)	63 (100.00)	0 (0.00)	
Low (6 h or less per week)	75 (39.89)	0 (0.00)	75 (60.00)	
High (more than 6 h per week)	50 (26.60)	0 (0.00)	50 (40.00)	
**Number of years of walking practice**				< 0.001^a^
Midlife (40–64 years)	18.18 (13.37)	0 (0.00)	18.18 (13.37)	
Late life (≥65 years)	4.95 (6.28)	0 (0.00)	4.95 (6.28)	
Overall	9.98 (13.02)	0 (0.00)	15.43 (13.35)	
**Walking onset**				< 0.001^b^
Midlife (40−64 years)	103 (54.79)	0 (0.00)	103 (82.40)	
Late life (≥65 years)	22 (11.70)	0 (0.00)	22 (17.60)	
**Annual income**, no. (%)				
< MCL	23 (12.23)	9 (14.29)	14 (11.20)	0.371^b^
≥ MCL, < 2×MCL	59 (31.38)	23 (36.51)	36 (28.80)	
≥ 2×MCL	106 (56.38)	31 (49.21)	75 (60.00)	
VRS, %	23.85 (18.57)	25.40 (17.67)	23.07 (19.02)	0.418^a^
**GDS, no. (%)**				0.572^b^
Normal (< 9)	92 (48.94)	29 (46.03)	63 (50.40)	
Depressed (≥10)	96 (51.06)	34 (53.97)	62 (49.60)	
UPDRS, gait disturbance requiring assistance	0 (0.00)	0 (0.00)	0 (0.00)	
**Body mass index**				
Underweight (< 18.5)	4 (2.13)	0 (0.00)	4 (3.20)	0.131^c^
Normal weight (18.5–24.9)	95 (50.53)	28 (44.44)	67 (53.60)	
Overweight (≥ 25)	89 (47.34)	35 (55.56)	54 (43.20)	
**Alcohol drink status, no (%)**				0.846^b^
Never	105 (55.85)	35 (55.56)	70 (56.00)	
Former	32 (17.02)	12 (19.05)	20 (16.00)	
Drinker	51 (27.13)	16 (25.40)	35 (28.00)	
PASE total score	65.56 (46.58)	70.73 954.79)	62.96 (41.84)	0.281^a^
**Blood markers**				
Hemoglobin	13.33 (1.52)	13.25 (1.60)	13.37 (1.49)	0.592^a^
Albumin	4.57 (0.26)	4.57 (0.26)	4.58 (0.26)	0.906^a^
Glucose, fasting	108.40 (20.31)	108.34 (17.76)	108.44 (21.54)	0.976^a^
HDL-cholesterol	54.87 (12.90)	53.58 (13.61)	55.52 (12.54)	0.336^a^
LDL-cholesterol	96.13 (33.40)	98.21 (34.64)	95.10 (32.85)	0.550^a^
**Nutritional markers**				
Protein, no (%)				0.390^b^
High	25 (13.30)	6 (9.52)	19 (15.20)	
Moderate	73 (38.83)	23 (36.51)	50 (40.00)	
Low	90 (47.87)	34 (53.97)	56 (44.80)	
Fruit and vegetables, no (%)				0.972^b^
High	128 (68.09)	43 (68.25)	85 (68.00)	
Low	60 (31.91)	20 (31.75)	40 (32.00)	
Decrease in food intake over the past 3 months, no (%)	14 (7.45)	6 (9.52)	8 (6.40)	0.557^c^
**CERAD cognition**				
Total score	70.50 (15.47)	65.21 (14.48)	73.17 (15.32)	< 0.001^a^
Memory score	35.45 (9.31)	32.56 (9.62)	36.91 (8.83)	0.002^a^
Non-memory score	34.39 (6.64)	33.05 (6.56)	35.07 (6.59)	0.048^a^

Data are expressed as mean (standard deviation), unless otherwise indicated

*MMSE* mini-mental status examination, *APOE4* apolipoprotein ε4, *CN* cognitively normal, *MCL* minimum cost of living, *VRS* vascular risk score, *GDS* geriatric depression scale, *UPDRS* Unified Parkinson’s Disease Rating Scale, *PASE* physical activity scale for the elderly, *CERAD* Consortium to Establish a Registry for Alzheimer’s Disease

^a^By Student *t*-test

^b^By chi-square test

^c^By Fisher exact test

**Table 2:** Baseline participant characteristics according to walking intensity and duration

	None	Walking intensity			Walking duration		
		Low	High	*p*	Low	High	*p*
*n*	63	68	57		75	50	
Age, years	72.67 (5.23)	72.24 (5.64)	71.00 (4.34)	0.189^a^	72.32 (5.39)	70.70 (4.52)	0.104^a^
Female, no. (%)	45 (71.43)	56 (82.35)	32 (56.14)	0.060^b^	57 (76.00)	31 (62.00)	0.237^b^
**Education, no. (%)**				0.074^b^			0.071^b^
≥13 years	9 (14.29)	12 (17.65)	18 (31.58)		16 (21.33)	14 (28.00)	
10–12 years	14 (22.22)	17 (25.00)	19 (33.33)		20 (26.67)	16 (32.00)	
4–9 years	33 (52.38)	33 (48.53)	16 (28.07)		29 (38.67)	20 (40.00)	
0–3 years	7 (11.11)	6 (8.82)	4 (7.02)		10 (13.33)	0 (0.00)	
MMSE	24.76 (3.80)	25.47 (3.64)	26.91 (2.19)	0.002^a^	25.65 (3.43)	26.84 (2.50)	0.005^a^
APOE4 positivity, No. (%)	13 (20.63)	16 (23.53)	8 (14.04)	0.402^b^	17 (22.67)	7 (14.00)	0.477^b^
Clinical diagnosis, CN, no. (%)	33 (52.38)	37 (54.41)	37 (64.91)	0.335^b^	43 (57.33)	31(62.00)	0.588^b^
**Walking onset**				< 0.001^b^			< 0.001^b^
Midlife (40–64 years)	0 (0.00)	58 (85.29)	45 (78.95)		62 (82.67)	41 (82.00)	
Late life (≥65 years)	0 (0.00)	10 (14.71)	12 (21.05)		13 (17.33)	9 (18.00)	
**Annual income**, no. (%)				0.111 ^b^			0.289^b^
< MCL	9 (14.29)	11 (16.18)	3 (5.26)		11 (14.67)	3 (6.00)	
≥ MCL, < 2×MCL	23 (36.51)	22 (32.35)	14 (24.56)		23 (30.67)	13 (26.00)	
≥ 2×MCL	31 (49.21)	35 (51.47)	40 (70.18)		41 (54.67)	34 (68.00)	
VRS, %	25.40 (17.67)	22.55 (17.70)	23.68 (20.64)	0.681^a^	22.89 (17.28)	23.33 (21.56)	0.715^a^
**GDS, no. (%)**				0.067^b^			0.817^b^
Normal (< 9)	29 (36.51)	28 (41.18)	35 (61.40)		37 (49.33)	26 (52.00)	
Depressed (≥10)	34 (53.97)	40 (58.82)	22 (38.60)		38 (50.67)	24 (48.00)	
UPDRS, gait score (>2)	0 (0.00)	0 (0.00)	0 (0.00)		0 (0.00)	0 (0.00)	
**Body mass index**				0.179^c^			0.085^c^
Underweight (< 18.5)	0 (0.00)	3 (4.41)	1 (1.75)		4 (5.33)	0 (0.00)	
Normal weight (18.5–24.9)	2 (44.44)	39 (57.35)	28 (49.12)		39 (52.00)	28 (56.00)	
Overweight (≥ 25)	35(55.55)	26 (38.24)	28 (49.12)		32 (42.67)	22 (44.00)	
**Alcohol drink status, no (%)**				0.832^b^			0.336^b^
Never	35 (55.56)	41 (60.29)	29 (50.88)		46 (61.33)	24 (48.00)	
Former	12	10	10		13	7	
Drinker	16	17	18		16	19	
PASE total score	70.73 (54.79)	61.44 (44.95)	64.76 (38.12)	0.518^a^	61.43 (45.67)	65.24 (35.65)	0.507^a^
**Blood markers**							
Hemoglobin	13.25 (1.60)	13.10 (1.58)	13.70 (1.31)	0.081^a^	13.38 (1.53)	13.37 (1.52)	0.865^a^
Albumin	4.57 (0.26)	4.57 (0.24)	4.59 (0.29)	0.898^a^	4.56 (0.24)	4.60 (0.29)	0.775^a^
Glucose, fasting	108.34 (17.76)	107.56 (21.24)	109.50 (22.04)	0.870^a^	108.31 (22.37)	108.62 (20.47)	0.996^a^
HDL-cholesterol	53.58 (13.61)	55.59 (13.61)	55.43 (11.24)	0.629^a^	55.20 (12.40)	55.98 (12.86)	0.598^a^
LDL-cholesterol	98.21 (34.64)	97.50 (34.01)	92.18 (31.45)	0.568^a^	96.34 (33.49)	93.26 (32.13)	0.738^a^
**Nutritional markers**							
Protein, no (%)				0.119^b^			0.475^b^
High	6 (9.52)	6 (13.24)	13 (22.81)		10 (13.33)	9 (18.00)	
Moderate	23 (36.51)	28 (41.18)	22 (38.60)		28 (37.33)	22 (44.00)	
Low	34 (53.97)	34 (51.47)	22 (38.60)		37 (49.33)	19 (38.00)	
Fruit and Vegetables, no (%)				0.689^b^			0.147^b^
High	43 (68.25)	44 (64.71)	41 (71.93)		46 (61.33)	39 (78.00)	
Low	20 (31.75)	24 (35.29)	16 (28.07)		29 (38.67)	11 (22.00)	
Decrease in food intake over the past 3 months, No (%)	6 (9.52)	4 (5.88)	4 (7.02)	0.722^c^	5 (6.67)	3 (6.00)	0.736^c^
**CERAD cognition**							
Total score	65.21 (14.48)	68.38 (14.40)	78.88 (14.51)	< 0.001^a^	71.29(15.85)	75.98 (14.17)	< 0.001^a^
Memory score	32.56 (9.62)	35.01 (9.77)	39.18 (7.00)	< 0.001^a^	36.20 (9.72)	37.98 (7.25)	0.005^a^
Non-memory score	33.05 (6.56)	33.18 (6.33)	37.33 (6.22)	< 0.001^a^	34.12 (6.99)	36.50 (5.73)	0.020^a^

Data are expressed as mean (standard deviation), unless otherwise indicated

*MMSE* mini-mental status examination, *APOE4* apolipoprotein ε4, *CN* cognitively normal, *MCL* minimum cost of living, *VRS* vascular risk score, *GDS* geriatric depression scale, *UPDRS* Unified Parkinson’s Disease Rating Scale, *PASE* physical activity scale for the elderly, *CERAD* Consortium to Establish a Registry for Alzheimer’s Disease

^a^By one-way analysis of variance

^b^By chi-square test

^c^By Fisher exact test

### Association between walking and cognition

**Fig. 1:**
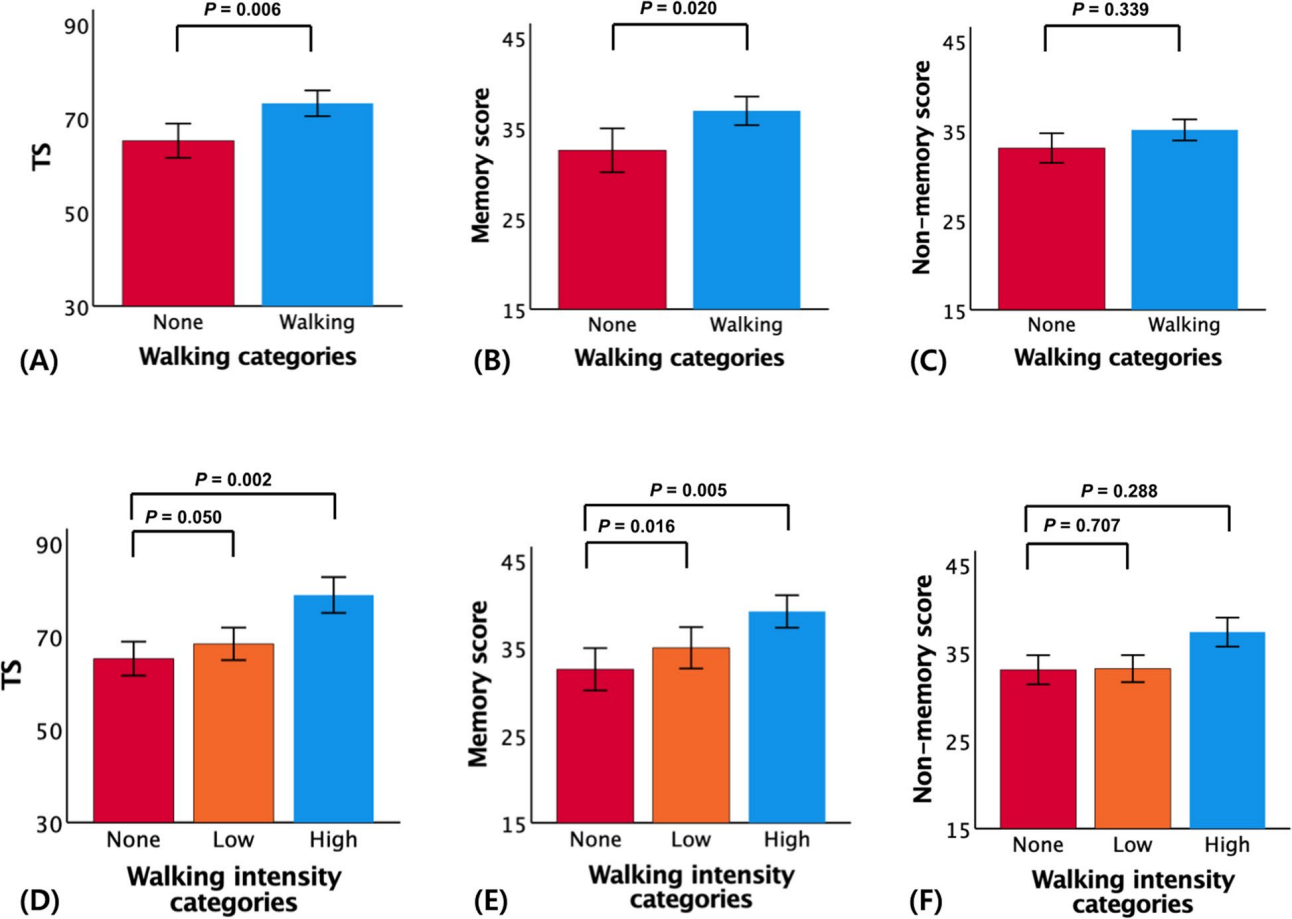
Plots of the associations between walking and cognition (**A**–**F**): **A** walking vs. total score (TS), **B** walking vs. memory score, **C** walking vs. non-memory score, **D** midlife-initiated walking intensity vs. TS, **E** midlife-initiated walking intensity vs. memory score, and **F** midlife-initiated walking intensity vs. non-memory score. **A**–**F** were adjusted for potential covariates; mean cognition values are presented and error bars represent standard error

The walking group showed better episodic memory (*B* = 2.852, *SE* = 1.214, *β* = 0.144, *p* = 0.020), but not non-memory cognition, compared to the non-walking group, even after controlling for the confounding factors. The walking group also showed better overall cognition (*B* = 5.477, *SE* = 1.984, *β* = 0.166, *p* = 0.006) compared to the non-walking group (Table 3 and Fig. 1).

**Table 3:** Results of multiple linear regression analyses of the associations between walking and cognition (*n* = 188)

	Total score				Memory score				Non-memory score			
	*B*	*SE*	$\beta$	*p*	*B*	*SE*	$\beta$	*p*	*B*	*SE*	$\beta$	*p*
Overall walking (*n* = 125)												
**Model 1**^a^												
Walking	7.056	2.223	0.214	**0.002**	3.647	1.310	0.185	**0.006**	1.794	1.004	0.127	0.076
None	Reference				Reference				Reference			
**Model 2**^b^												
Walking	5.477	1.984	0.166	**0.006**	2.852	1.214	0.144	**0.020**	0.889	0.063	0.063	0.339
None	Reference				Reference				Reference			
Midlife-initiated walking (*n* = 103)												
**Model 1**^a^												
Walking	7.496	2.425	0.226	**0.002**	4.016	1.412	0.207	**0.005**	1.931	1.080	0.137	0.076
None	Reference				Reference				Reference			
**Model 2**^b^												
Walking	5.439	2.156	0.164	**0.013**	2.936	1.313	0.152	**0.027**	0.848	0.990	0.060	0.393
None	Reference				Reference				Reference			
Late life-initiated walking (*n* = 22)												
**Model 1**^a^												
Walking	5.541	3.255	0.177	0.093	2.418	2.213	0.114	0.278	1.672	1.556	0.116	0.286
None	Reference				Reference				Reference			
**Model 2**^b^												
Walking	5.927	3.120	0.189	0.062	3.173	2.176	0.149	0.150	1.072	1.586	0.075	0.501
None	Reference				Reference				Reference			

For these analyses, we used the no walking (*n* = 63) as the reference

*APOE4* apolipoprotein ε4, *GDS* geriatric depression scale, *VRS* vascular risk score, *BMI* body mass index

^a^Adjusted for age, sex, and APOE4

^b^Adjusted for covariates in model 1 plus, education, clinical diagnosis, GDS, annual income, alcohol intake, smoking, VRS, BMI, dietary pattern including food types (such as protein and fruit or vegetables), serum nutritional markers (such as hemoglobin, albumin, glucose, and HDL-/LDL-cholesterol), and overall physical activity score

### Associations of walking intensity and duration with cognition

The high intensity group showed better episodic memory (*B* = 6.743, *SE* = 2.887, *β* = 0.334, *p* = 0.021) and overall cognition (*B* = 12.729, *SE* = 4.664, *β* = 0.377, *p* = 0.007), but not non-memory cognition, compared to the non-walking group (Table 4). By contrast, there was no difference in cognition between the walking duration groups (Table 5).

**Table 4:** Results of multiple linear regression analyses of the association between walking intensity and cognition (*n* = 188)

	Total score				Memory score				Non-memory score			
	*B*	*SE*	$\beta$	*p*	*B*	*SE*	$\beta$	*p*	*B*	*SE*	$\beta$	*p*
Overall walking (*n* = 125)												
**Model 1**^a^												
High	11.839	2.613	0.350	**< 0.001**	5.180	1.573	0.256	**0.001**	3.650	1.189	0.252	**0.002**
Low	3.369	2.441	0.105	0.169	2.466	1.469	0.128	0.095	0.363	1.111	0.026	0.744
None	Reference				Reference				Reference			
**Model 2**^b^												
High	8.693	2.390	0.257	**< 0.001**	3.797	0.188	0.188	**0.011**	2.011	1.126	0.139	0.076
Low	3.229	2.180	0.100	0.140	2.191	1.351	0.114	0.107	0.104	1.027	0.008	0.919
None	Reference				Reference				Reference			
**Model 3**^c^												
High	12.729	4.664	0.377	**0.007**	6.743	2.887	0.334	**0.021**	2.054	2.204	0.142	0.353
Low	6.279	3.730	0.195	0.094	4.418	2.309	0.230	0.057	0.136	1.763	0.010	0.939
None	Reference				Reference				Reference			
Midlife-initiated walking (*n* = 103)												
**Model 1**^a^												
High	12.960	2.901	0.360	**< 0.001**	5.675	1.729	0.271	**0.001**	3.863	1.308	0.253	**0.004**
Low	3.459	2.668	0.103	0.197	2.790	1.590	0.143	0.081	0.503	1.203	0.035	0.676
None	Reference				Reference				Reference			
**Model 2**^b^												
High	9.390	2.671	0.261	**< 0.001**	3.949	1.654	0.188	**0.018**	1.910	1.243	0.125	0.127
Low	2.902	2.363	0.086	0.221	2.286	1.463	0.117	0.120	0.166	1.100	0.012	0.880
None	Reference				Reference				Reference			
**Model 3**^c^												
High	16.624	5.373	0.462	**0.002**	9.360	3.314	0.446	**0.005**	2.686	2.570	0.176	0.288
Low	8.175	4.138	0.244	0.050	6.231	2.552	0.318	**0.016**	0.732	1.941	0.051	0.707
None	Reference				Reference				Reference			
Late life-initiated walking (*n* = 22)												
**Model 1**^a^												
High	7.700	4.284	0.192	0.076	3.736	2.915	0.137	0.204	3.244	2.036	0.176	0.115
Low	3.067	4.557	0.073	0.503	0.907	3.101	0.032	0.771	− 0.131	2.166	− 0.007	0.952
None	Reference				Reference				Reference			
**Model 2**^b^												
High	9.947	4.272	0.247	**0.023**	5.833	2.983	0.214	0.055	3.225	2.167	0.175	0.142
Low	1.705	4.374	0.041	0.698	0.379	3.055	0.013	0.902	− 1.190	2.220	− 0.062	0.594
None	Reference				Reference				Reference			
**Model 3**^c^												
High	− 4.518	9.567	− 0.112	0.638	− 4.244	6.681	− 0.156	0.528	− 3.200	4.880	− 0.174	0.514
Low	− 12.085	9.255	− 0.289	0.196	− 9.228	6.464	− 0.325	0.158	− 7.315	4.721	− 0.382	0.126
None	Reference				Reference				Reference			

For these analyses, we used the no walking (*n* = 63) as the reference

*APOE4*, apolipoprotein ε4; *GDS*, geriatric depression scale; *VRS*, vascular risk score; *BMI*, body mass index

^a^Adjusted for age, sex, and APOE4

^b^Adjusted for covariates in model 1 plus, education, clinical diagnosis, GDS, annual income, alcohol intake, smoking, VRS, BMI, dietary pattern including food types (such as protein and fruit or vegetables), serum nutritional markers (such as hemoglobin, albumin, glucose, and HDL-/LDL-cholesterol), and overall physical activity score

^c^Adjusted for covariates in model 2 plus, walking duration

**Table 5:** Results of multiple linear regression analyses of the association between walking duration and cognition (*n* = 188)

	Total score				Memory score				Non-memory score			
	*B*	*SE*	$\beta$	*p*	*B*	*SE*	$\beta$	*p*	*B*	*SE*	$\beta$	*p*
Overall (*n* = 125)												
**Model 1**^a^												
High	8.482	2.748	0.243	**0.002**	3.816	1.623	0.183	**0.020**	2.734	1.238	0.182	**0.028**
Low	6.151	2.449	0.194	**0.013**	3.540	1.446	0.187	**0.015**	1.197	1.103	0.088	0.279
None	Reference				Reference				Reference			
**Model 2**^b^												
High	5.495	2.177	0.173	**0.013**	3.210	1.331	0.169	**0.017**	0.622	1.017	0.046	0.542
Low	5.447	2.460	0.156	**0.028**	2.266	1.503	0.108	0.134	1.326	1.149	0.088	0.250
None	Reference				Reference				Reference			
**Model 3**^c^												
High	− 2.676	3.851	− 0.084	0.488	0.264	2.980	0.014	0.912	− 1.808	1.820	− 0.133	0.322
Low	− 5.181	4.815	− 0.148	0.283	− 1.565	2.384	− 0.075	0.600	− 1.834	2.275	− 0.122	0.421
None	Reference				Reference				Reference			
Midlife-initiated walking (*n* = 103)												
**Model 1**^a^												
High	6.958	2.695	0.210	**0.011**	4.318	1.569	0.224	**0.007**	1.429	1.198	0.102	0.235
Low	8.353	3.058	0.225	**0.007**	3.534	1.780	0.164	**0.049**	2.730	1.359	0.174	**0.046**
None	Reference				Reference				Reference			
**Model 2**^b^												
High	5.768	2.371	0.174	**0.016**	3.673	1.437	0.190	**0.012**	0.722	1.089	0.051	0.508
Low	4.877	2.727	0.132	0.076	1.677	1.653	0.078	0.312	1.064	1.253	0.068	0.397
None					Reference							
**Model 3**^c^	Reference								Reference			
High	− 4.684	4.311	− 0.142	0.279	− 0.197	2.659	− 0.010	0.941	− 1.695	2.022	− 0.121	0.403
Low	− 9.096	5.544	− 0.245	0.103	− 3.497	3.419	− 0.162	0.308	− 2.168	2.601	− 0.138	0.406
None	Reference				Reference				Reference			
Late life-initiated walking (*n* = 22)												
**Model 1**^a^												
High	9.811	4.598	0.224	**0.036**	5.929	3.110	0.200	0.060	3.335	2.206	0.166	0.135
Low	2.191	4.130	0.057	0.569	− 0.337	2.793	− 0.013	0.904	0.366	1.981	0.021	0.854
None	Reference				Reference				Reference			
**Model 2**^b^												
High	11.636	4.460	0.265	**0.011**	7.139	3.110	0.240	**0.025**	3.636	2.279	0.181	0.116
Low	1.354	4.018	0.035	0.737	− 0.005	2.802	< 0.001	0.999	− 0.981	2.053	− 0.055	0.634
None	Reference				Reference				Reference			
**Model 3**^c^												
High	− 0.084	10.234	− 0.002	0.993	− 0.580	7.147	− 0.020	0.936	− 2.738	5.220	− 0.136	0.602
Low	− 9.868	9,693	− 0.255	0.313	− 7.396	6.769	− 0.282	0.279	− 7.084	4.944	− 0.399	0.157
None	Reference				Reference				Reference			

For these analyses, we used the no walking (*n* = 63) as the reference

*APOE4* Apolipoprotein ε4, *GDS* Geriatric depression scale, *VRS* Vascular risk score, *BMI* Body mass index

^a^Adjusted for age, sex, and APOE4

^b^Adjusted for covariates in model 1 plus, education, clinical diagnosis, GDS, annual income, alcohol intake, smoking, VRS, BMI, dietary pattern including food types (such as protein and fruit or vegetables), serum nutritional markers (such as hemoglobin, albumin, glucose, and HDL-/LDL-cholesterol), and overall physical activity score

^c^Adjusted for covariates in model 2 plus, walking intensity

### Associations of midlife- and late life-initiated walking with cognition

Midlife-initiated (before 65 years of age) walking as well as walking intensity were significantly associated with better episodic memory (*B* = 2.936, *SE* = 1.313, *β* = 0.152, *p* = 0.027 in the walking group; *B* = 9.360, *SE* = 3.314, *β* = 0.446, *p* = 0.005 in the high-intensity walking group) and overall cognition (*B* = 5.439, *SE* = 2.156, *β* = 0.164, *p* = 0.013 in the walking group; *B* = 16.624, *SE* = 5.373, *β* = 0.462, *p* = 0.002 in the high-intensity walking group) (Tables 3 and 4; Fig. 1). By contrast, there was no difference in cognition according to walking duration, regardless of the time of walking initiation (Table 5).

### Moderation of the association between walking intensity and cognition

The association between walking intensity and cognition was not significantly moderated by age, sex, APOE4, education, clinical diagnosis, GDS score, annual income, VRS, or PASE total score (Table 6).

**Table 6:** Results of multiple linear regression analyses including walking intensity $\times$ one covariate interaction term, predicting cognition (*n* = 188)

	Total score				Memory score				Non-memory score			
	*B*	*SE*	$\beta$	*p*	*B*	*SE*	$\beta$	*p*	*B*	*SE*	$\beta$	*p*
High-intensity walking	41.256	35.464	1.221	0.246	29.587	21.785	1.465	0.176	1.844	16.789	0.127	0.913
Low-intensity walking	18.925	29.918	0.588	0.528	36.497	18.378	1.897	0.049	− 2.501	14.164	− 0.181	0.860
Age	− 0.325	0.308	− 0.108	0.293	− 0.079	0.189	− 0.044	0.675	− 0.152	0.146	− 0.118	0.300
High-intensity walking $\times$ age	− 0.397	0.490	− 0.836	0.419	− 0.314	0.301	− 1.105	0.299	0.002	0.232	0.012	0.992
Low-intensity walking $\times$ age	− 0.172	0.408	− 0.389	0.673	− 0.440	0.250	− 1.661	0.081	0.036	0.193	0.191	0.851
High-intensity walking	13.494	4.910	0.399	0.007	7.691	3.042	0.381	0.012	1.135	2.309	0.078	0.624
Low-intensity walking	4.739	3.888	0.147	0.225	3.822	2.409	0.199	0.114	− 0.980	1.828	− 0.071	0.593
Sex	− 6.349	4.105	− 0.186	0.124	− 2.415	2.544	− 0.118	0.344	− 3.857	1.931	− 0.263	0.047
High-intensity walking $\times$ sex	− 1.580	4.979	− 0.035	0.751	− 2.572	3.085	− 0.095	0.406	3.286	2.342	0.169	0.162
Low-intensity walking $\times$ sex	7.042	5.303	0.112	0.186	2.658	3.286	0.071	0.420	3.252	2.686	0.120	0.228
High-intensity walking	14.601	4.860	0.432	0.003	7.501	2.997	0.371	0.013	2.572	2.299	0.178	0.265
Low-intensity walking	7.768	3.888	0.241	0.047	5.466	2.408	0.284	0.024	0.468	1.847	0.034	0.800
APOE4	− 2.730	3.969	− 0.069	0.493	− 2.869	2.457	− 0.121	0.245	− 0.142	1.885	− 0.008	0.940
High-intensity walking $\times$ APOE4	− 9.706	6.496	− 0.119	0.137	− 4.329	4.022	− 0.089	0.283	− 2.615	3.085	− 0.075	0.398
Low-intensity walking $\times$ APOE4	− 5.650	5.292	− 0.102	0.287	− 4.697	3.277	− 0.142	0.154	− 1.128	2.514	− 0.048	0.654
High-intensity walking	10.886	6.236	0.322	0.083	8.570	3.856	0.424	0.028	0.193	2.931	0.013	0.948
Low-intensity walking	4.164	5.151	0.129	0.420	4.065	3.185	0.211	0.204	− 2.208	2.421	− 0.160	0.363
Education	4.509	2.006	0.267	0.026	3.377	1.241	0.335	0.007	1.041	0.943	0.144	0.271
High-intensity walking $\times$ education	1.258	2.584	0.082	0.627	− 1.074	1.598	− 0.118	0.502	1.285	1.215	0.196	0.292
Low-intensity walking $\times$ education	1.549	2.519	0.090	0.540	0.146	1.558	0.014	0.926	1.707	1.184	0.232	0.151
High-intensity walking	10.830	4.408	0.320	0.015	4.375	2.307	0.217	0.060	1.723	1.425	0.119	0.229
Low-intensity walking	4.811	3.686	0.149	0.194	3.101	1.929	0.161	0.110	− 0.371	1.395	− 0.027	0.791
Clinical diagnosis	− 14.080	2.743	− 0.449	< 0.001	− 12.049	1.436	− 0.643	< 0.001	− 2.957	1.475	− 0.220	0.047
High-intensity walking $\times$ clinical diagnosis	1.335	4.166	0.027	0.749	3.066	2.181	0.103	0.162	− 0.051	2.238	− 0.002	0.982
Low-intensity walking $\times$ clinical diagnosis	1.656	3.823	0.040	0.665	1.429	2.001	0.058	0.476	0.734	2.056	0.041	0.722
High-intensity walking	11.353	5.205	0.336	0.031	5.167	3.213	0.256	0.110	1.619	2.454	0.112	0.510
Low-intensity walking	5.852	4.522	0.182	0.197	3.287	2.792	0.171	0.241	0.818	2.132	0.059	0.702
GDS	− 5.027	3.211	− 0.162	0.119	− 2.381	1.982	− 0.128	0.231	− 1.961	1.514	− 0.147	0.197
High-intensity walking $\times$ GDS	2.954	4.736	0.062	0.534	3.258	2.923	0.114	0.267	1.095	2.233	0.053	0.624
Low-intensity walking $\times$ GDS	0.796	4.469	0.021	0.859	2.035	2.759	0.090	0.462	− 1.176	2.107	− 0.073	0.578
High-intensity walking	27.001	9.966	0.799	0.007	13.000	6.194	0.644	0.037	5.042	4.748	0.348	0.290
Low-intensity walking	4.310	7.702	0.134	0.576	2.589	4.787	0.135	0.589	− 0.795	3.669	− 0.058	0.829
Annual income	4.410	2.279	0.200	0.055	2.528	1.417	0.192	0.076	2.225	1.086	0.238	0.039
High-intensity walking $\times$ annual income	− 5.698	3.634	− 0.461	0.119	− 2.470	2.259	− 0.334	0.276	− 1.178	1.731	− 0.222	0.497
Low-intensity walking $\times$ annual income	0.678	2.975	0.053	0.820	0.717	1.849	0.094	0.699	0.367	1.417	0.067	0.796
High-intensity walking	9.116	5.437	0.270	0.095	5.797	3.377	0.287	0.088	− 0.234	2.558	− 0.016	0.927
Low-intensity walking	4.535	4.948	0.141	0.361	2.771	3.073	0.144	0.369	− 1.004	2.328	− 0.073	0.667
VRS	− 0.049	0.093	− 0.059	0.598	− 0.044	0.058	− 0.089	0.446	− 0.019	0.044	− 0.052	0.669
High-intensity walking $\times$ VRS	0.163	0.121	0.165	0.181	0.032	0.075	0.054	0.674	0.103	0.057	0.244	0.073
Low-intensity walking $\times$ VRS	0.074	0.123	0.073	0.548	0.062	0.076	0.102	0.415	0.048	0.058	0.110	0.406
High-intensity walking	16.931	5.682	0.501	0.003	9.787	3.503	0.485	0.006	4.966	2.669	0.343	0.065
Low-intensity walking	6.979	4.750	0.217	0.144	4.066	2.929	0.211	0.167	0.956	2.231	0.069	0669
Overall physical activity	0.068	0.029	0.203	0.021	0.036	0.018	0.182	0.044	0.025	0.014	0.177	0.065
High-intensity walking $\times$ overall PA	− 0.071	0.054	− 0.164	0.190	− 0.053	0.033	− 0.204	0.114	− 0.018	0.023	− 0.097	0.427
Low-intensity walking $\times$ overall PA	− 0.015	0.045	− 0.038	0.744	0.002	0.028	0.009	0.940	0.007	0.017	0.043	0.680

*APOE4* apolipoprotein ε4, *GDS* Geriatric depression scale, *VRS* Vascular risk score, *BMI* Body mass index, *PA* Physical activity

### Sensitivity analyses

The older individuals exhibiting no decrease in food intake over the past 3 months had similar cognitive performance to the entire cohort (Table 7).

**Table 7:** Results of multiple linear regression analyses of the association between walking (or walking intensity) and cognition in older adults exhibiting no decrease in food intake over the past 3 months (*n* = 174)

Walking	Total score				Memory score				Non-memory score			
	*B*	*SE*	$\beta$	*p*	*B*	*SE*	$\beta$	*p*	*B*	*SE*	$\beta$	*p*
Overall walking (*n* = 117)												
**Model 1**^a^												
Walking	6.408	2.276	0.200	**0.005**	3.863	1.309	0.202	**0.004**	1.253	1.044	0.090	0.232
None	Reference				Reference				Reference			
**Model 2**^b^												
Walking	5.124	2.043	0.160	**0.013**	3.133	1.204	0.164	**0.010**	0.431	0.979	0.031	0.661
None	Reference				Reference				Reference			
Midlife-initiated walking (*n* = 97)												
**Model 1**^a^												
Walking	6.947	2.484	0.217	**0.006**	4.282	1.407	0.230	**0.003**	1.477	1.120	0.107	0.189
None	Reference				Reference				Reference			
**Model 2**^b^												
Walking	5.084	2.217	0.158	**0.023**	3.180	1.295	0.171	**0.015**	0.393	1.045	0.029	0.707
None	Reference				Reference				Reference			
Late life-initiated walking (*n* = 20)												
**Model 1**^a^												
Walking	4.293	3.307	0.146	0.199	2.212	2.210	0.111	0.321	0.965	1.557	0.072	0.538
None	Reference				Reference				Reference			
**Model 2**^b^												
Walking	4.956	3.122	0.168	0.118	3.468	2.102	0.173	0.105	0.432	1.567	0.032	0.784
None	Reference				Reference				Reference			
Walking intensity	Total score				Memory score				Non-memory score			
	B	SE	$\beta$	*p*	B	SE	$\beta$	*p*	B	SE	$\beta$	*p*
Overall walking (*n* = 117)												
**Model 1**^a^												
High	11.083	2.618	0.344	**< 0.001**	4.533	1.562	0.237	**0.004**	3.222	1.203	0.232	**0.008**
Low	2.901	2.417	0.094	0.232	2.273	1.442	0.125	0.117	− 0.020	1.111	− 0.001	0.986
None	Reference				Reference				Reference			
**Model 2**^b^												
High	8.349	2.400	0.260	**< 0.001**	3.162	1.454	0.165	**0.031**	1.755	1.168	0.126	0.135
Low	3.143	2.164	0.102	0.148	2.197	1.311	0.120	0.096	− 0.154	1.053	− 0.012	0.884
None	Reference				Reference				Reference			
**Model 3**^c^												
High	12.757	4.622	0.397	**0.006**	6.587	2.794	0.345	**0.020**	1.834	2.258	0.132	0.418
Low	6.477	3.689	0.211	0.081	4.787	2.230	0.262	**0.033**	− 0.095	1.802	− 0.007	0.958
None	Reference				Reference				Reference			
Midlife-initiated walking (*n* = 97)												
**Model 1**^a^												
High	12.520	2.892	0.367	**< 0.001**	5.229	1.708	0.266	**0.003**	3.573	1.315	0.246	**0.007**
Low	3.093	2.638	0.096	0.243	2.645	1.558	0.143	0.092	0.182	1.200	0.013	0.880
None	Reference				Reference				Reference			
**Model 2**^b^												
High	9.317	2.676	0.273	**< 0.001**	3.388	1.617	0.172	**0.038**	1.751	1.294	0.121	0.178
Low	2.908	2.332	0.091	0.215	2.314	1.409	0.125	0.103	− 0.043	1.128	− 0.003	0.970
None	Reference				Reference				Reference			
**Model 3**^c^												
High	17.505	5.322	0.514	**0.001**	9.569	3.195	0.486	**0.003**	2.697	2.602	0.186	0.302
Low	8.842	4.065	0.276	**0.031**	6.793	2.440	0.367	**0.006**	0.642	1.988	0.047	0.747
None	Reference				Reference				Reference			
Late life-initiated walking (*n* = 20)												
**Model 1**^a^												
High	4.809	4.327	0.123	0.270	1.713	3.021	0.064	0.572	2.226	2.059	0.125	0.283
Low	2.428	4.370	0.062	0.580	0.541	3.051	0.020	0.860	− 0.481	2.079	0.125	0.283
None	Reference				Reference				Reference			
**Model 2**^b^												
High	8.306	4.208	0.213	0.053	5.011	2.968	0.186	0.097	2.126	2.253	0.119	0.349
Low	1.771	4.100	0.045	0.667	0.135	2.892	0.005	0.963	− 1.073	2.196	− 0.060	0.627
None	Reference				Reference				Reference			
**Model 3**^c^												
High	− 6.584	8.739	− 0.169	0.454	− 5.572	6.161	− 0.207	0.370	− 4.082	4.741	− 0.229	0.393
Low	− 13.185	8.720	− 0.338	0.136	− 10.494	6.148	− 0.389	0.093	− 7.308	4.731	− 0.411	0.128
None	Reference				Reference				Reference			

For these analyses, we used the no walking (*n* = 57) as the reference

*APOE4* Apolipoprotein ε4, *GDS* Geriatric depression scale, *VRS* Vascular risk score, *BMI* Body mass index

^a^Adjusted for age, sex, and APOE4

^b^Adjusted for covariates in Model 1 plus, education, clinical diagnosis, GDS, annual income, alcohol intake, smoking, VRS, BMI, dietary pattern including food types (such as protein and fruit or vegetables), serum nutritional markers (such as hemoglobin, albumin, glucose, and HDL-/LDL-cholesterol) and overall physical activity score

^c^Adjusted for covariates in Model 2 plus, walking duration

## Discussion

In the present study of physically capable older adults without dementia, the walking group had better episodic memory but not non-memory cognition, compared to the non-walking group. In particular, high-intensity walking was associated with better episodic memory, but not non-memory cognition. By contrast, there was no difference in cognition according to walking duration. The association between walking and better episodic memory was prominent in those who started walking in midlife (< 65 years of age).

The association between walking and episodic memory, which is the first cognitive domain to show a change in AD [3–6], seen in this study is in line with the results of previous studies demonstrating that walking prevents against AD development and cognitive decline [16, 17, 35]. The Study of Osteoporotic Fractures [16] showed that older women with greater baseline walking activity, measured as blocks walked or total kilocalories expended per week, were less likely to show a decline in the Mini-Mental State Examination score over the 6–8-year follow-up. The Honolulu-Asia Aging study [17] suggested that physically capable elderly men who walk regularly are less likely to develop AD. In particular, the risk of AD was 2.2-fold higher in men who walked < 0.25 miles per day compared to those who walked > 2 miles per day. The Harvard Aging Brain Study [35] showed that greater walking activity was associated with slower beta-amyloid peptide (Aβ)-related cognitive decline and neurodegeneration in asymptomatic older adults. However, that study assessed current walking activity (steps per day) using pedometers worn for 1 week; it did not assess lifetime walking activity (intensity, duration, and time of initiation), overall physical activity, or nutritional profiles. We found that high-intensity, midlife-initiated walking was associated with episodic memory, i.e., AD-related cognitive decline. Additionally, there were no moderating effects on the association between walking and cognition of potential covariates.

The mechanism underlying the protective effect of walking against AD-related cognitive decline remains unclear. It is difficult to clearly distinguish the mechanistic differences between walking and other exercises. Walking as physical activity regulates amyloid levels directly or indirectly through the moderation of gene products at the mRNA and protein levels; the associated anatomical, neurochemical, and electrophysiological changes promote neuronal plasticity [36]. Exercise directly modulates amyloid precursor protein (APP) metabolism [37] by increasing neuronal activity [38]. Exercise-induced upregulation of proteasome activity [39] may also mediate the degradation of proteolytic fragments of APP [40, 41]. In addition, cholinergic activity increases with exercise, and regulation of the cholinergic system has been implicated in exercise-induced plasticity [36]. Taken together, these findings support that neuronal activity may underlie the exercise-mediated regulation of APP processing. Further research is required to verify the mechanism by which walking protects against AD pathology, i.e., Aβ deposition.

In addition to these mechanisms, walking has a number of advantages that are different from other forms of exercises. Walking is the most sustainable voluntary physical activity and has no learning requirements. As walking accounts for a large proportion of lifetime physical activity, it may be particularly important with respect to the benefits of increased physical activity. For example, both aerobic walking and social dance have been found to improve white matter plasticity compared to active controls [42]. Notably, aerobic walking may be more beneficial than social dance with regard to white matter plasticity correlated with episodic memory [42]. In that study, dance classes included a significant amount of low-intensity instructional time, which may explain the lower benefits with regard to aerobic fitness [42].

In addition to walking, other physical activities may affect AD-related cognitive decline. The Cardiovascular Risk Factors, Aging and Incidence of Dementia study [28, 43] found that the performance of physical activity at least twice a week was associated with reduced risks of AD and dementia. Moreover, the Atherosclerosis Risk in Communities study [43] found that a high level of physical activity was associated with a lower incidence of dementia and lower decline. However, our results did not change after controlling for overall physical activity, indicating that high levels of walking activity are associated with cognitive performance independent of other physical activities and the overall activity level.

Unlike high-intensity walking, low-intensity walking was not associated with cognitive performance in this study. Additionally, cognition did not differ according to walking duration. Few studies have investigated the associations of the intensity and duration of walking with AD-related cognitive decline. Our finding of an association between high-intensity walking and cognitive performance is consistent with physical activity guidelines for older adults that recommend regular moderate-intensity walking or vigorous-intensity activity to achieve health benefits [27, 44]. We did not observe any difference regarding the benefits of walking for cognitive performance according to walking duration, in contrast to previous studies [17, 45, 46] showing a clear difference in the risk of dementia according to walking duration. The discrepancy between the results of the present and previous studies may be explained by the fact that maximal oxygen uptake only improves with higher-intensity physical activity [47]. Additionally, several studies have shown that the relative intensity, rather than duration, of physical activity was most important in terms of the effects on cardiovascular function [48] and cognition [28, 49–51]. Nevertheless, there requires a caution in interpretation of our findings regarding walking duration. This is because walking activity requires minimum walking frequency and duration as requirements for walking activity regardless of walking intensity.

We found significant associations between walking initiated in midlife, but not late life, and cognitive performance. This is consistent with a previous study that found that vigorous midlife physical activity lowered the AD risk [28] and was associated with reduced cognitive impairment [51]. This may be because participants who started walking in midlife had a longer duration thereof than those who started walking in later life (Table 1: mean [standard deviation] of number of years of walking practice: 18.2 [13.4] years in midlife walking onset group vs. 5.0 [6.3] years in late life onset group, *p* < 0.001). It may have also been due to the age of walking onset, as the same number of years of practice has different effects in midlife vs. late life. Therefore, we first examined any association between the number of years of walking practice and cognition to determine whether there was an association between the two (see Additional file 1: Table S1). Then, we examined the same model but including an interaction between age and the number of years of walking practice to examine whether the association between age and cognition was moderated by the number of years of walking practice (see Additional file 1: Table S2). There was only a slight moderation effect (see Additional file 1: Table S2: number of years of walking practice × age: *B* =  − 0.0021, *SE* = 0.012, *β* =  − 0.1296, *p* = 0.086 in total score; *B* =  − 0.015, *SE* = 0.009, *β* =  − 0.5276, *p* = 0.088 in memory score). However, we found no association between the number of years of walking practice and cognition (see Additional file 1: Table S1). Taken together, these observations suggest that the association between walking in midlife (but not late life) and cognition may be due to the age of walking onset rather than the number of years of walking practice.

In the present study, a comprehensive clinical assessment of physically capable non-demented older adults was performed covering lifetime walking activity (including intensity, duration, and time of initiation), overall physical activity level, laboratory blood tests, nutritional markers, and tests of multiple cognitive domains. We also controlled for potential confounders using statistical models investigating the association between walking and AD-related cognitive decline. However, there were several limitations to our study. First, this is a cross-sectional study which has limitations with respect to bi-directionality. Inferences on the causal relationships are constrained. There is a need for replication of the study findings in adequately powered prospective or trial studies to make inferences on the beneficial effects of walking on AD-related cognitive function. Second, walking may be limited by pre-existing brain diseases because it is a complex motor function that requires coordination of several body parts and gait [33]. To investigate the effect of walking on AD-related cognitive decline, we enrolled participants who were physically capable, i.e., able to walk without assistance (UPDRS gait score ≤ 2). Third, retrospective recall bias may have affected the association between lifetime walking and cognition. Approximately 40% of the participants were diagnosed with MCI, which may have led to inaccurate self-reported walking history. However, although individuals with MCI exhibit recent memory impairments, their remote memory is well-preserved [52]. Therefore, it is unlikely that our MCI participants reported inaccurate walking histories because such self-reports rely mainly on remote memory based on long-established lifestyle habits, rather than on recent memory. Additionally, we obtained similar results after controlling for clinical diagnosis (CN vs. MCI) as an additional covariate in model 2. Finally, we did not measure lifetime walking activity using an objective tool, unlike a previous study that evaluated physical activity [35] over a short period (1 week). Additionally, we did not assess lifetime physical activity in terms of duration or intensity. Further investigations using objective assessments of walking are needed to confirm the association between walking and cognition.

## Conclusions

The present study of physically capable older adults without dementia indicated that walking (particular in high-intensity and midlife-initiated walking) is associated with improved episodic memory, an AD-related cognitive domain. In terms of AD prevention, more attention needs to be paid to the role of walking.

### Supplementary Information

**Additional file 1.**

## Data Availability

The study data are not freely accessible because the IRB of the Hallym University Dongtan Sacred Heart Hospital prevents public sharing of such data for privacy reasons. However, the data are available on reasonable request after IRB approval. Requests for data access can be submitted to an independent administrative coordinator by e-mail (yoon4645@gmail.com).

## References

[1] Hardy J, Selkoe DJ (2002). The amyloid hypothesis of Alzheimer’s disease: progress and problems on the road to therapeutics. Science.

[2] Wilson RS, Segawa E, Boyle PA, Anagnos SE, Hizel LP, Bennett DA (2012). The natural history of cognitive decline in Alzheimer’s disease. Psychol Aging.

[3] Howieson DB, Dame A, Camicioli R, Sexton G, Payami H, Kaye JA (1997). Cognitive markers preceding Alzheimer’s dementia in the healthy oldest old. J Am Geriatr Soc.

[4] Backman L, Small BJ, Fratiglioni L (2001). Stability of the preclinical episodic memory deficit in Alzheimer’s disease. Brain.

[5] Backman L, Jones S, Berger AK, Laukka EJ, Small BJ (2005). Cognitive impairment in preclinical Alzheimer’s disease: a meta-analysis. Neuropsychology.

[6] Lim YY, Kong J, Maruff P, Jaeger J, Huang E, Ratti E (2022). Longitudinal cognitive decline in patients with mild cognitive impairment or dementia due to Alzheimer’s disease. J Prev Alzheimers Dis.

[7] Albert MS, Jones K, Savage CR, Berkman L, Seeman T, Blazer D (1995). Predictors of cognitive change in older persons: MacArthur studies of successful aging. Psychol Aging.

[8] Lautenschlager NT, Cox KL, Flicker L, Foster JK, van Bockxmeer FM, Xiao J (2008). Effect of physical activity on cognitive function in older adults at risk for Alzheimer disease: a randomized trial. JAMA.

[9] Ruiz-Muelle A, Lopez-Rodriguez MM (2019). Dance for people with Alzheimer’s disease: a systematic review. Curr Alzheimer Res.

[10] Abasiyanik Z, Yigit P, Ozdogar AT, Kahraman T, Ertekin O, Ozakbas S (2021). A comparative study of the effects of yoga and clinical Pilates training on walking, cognition, respiratory functions, and quality of life in persons with multiple sclerosis: a quasi-experimental study. Explore (NY).

[11] Mayor S (2015). Slow walking speed may be early sign of Alzheimer’s in older people, study finds. BMJ.

[12] Welmer AK, Rizzuto D, Qiu C, Caracciolo B, Laukka EJ (2014). Walking speed, processing speed, and dementia: a population-based longitudinal study. J Gerontol A Biol Sci Med Sci.

[13] Hackett RA, Davies-Kershaw H, Cadar D, Orrell M, Steptoe A (2018). Walking speed, cognitive function, and dementia risk in the English longitudinal study of ageing. J Am Geriatr Soc.

[14] Toots A, Littbrand H, Holmberg H, Nordstrom P, Lundin-Olsson L, Gustafson Y (2017). Walking aids moderate exercise effects on gait speed in people with dementia: a randomized controlled trial. J Am Med Dir Assoc.

[15] Toots A, Lundin-Olsson L, Nordstrom P, Gustafson Y, Rosendahl E (2021). Exercise effects on backward walking speed in people with dementia: a randomized controlled trial. Gait Posture.

[16] Yaffe K, Barnes D, Nevitt M, Lui LY, Covinsky K (2001). A prospective study of physical activity and cognitive decline in elderly women: women who walk. Arch Intern Med.

[17] Abbott RD, White LR, Ross GW, Masaki KH, Curb JD, Petrovitch H (2004). Walking and dementia in physically capable elderly men. JAMA.

[18] Morris JC (1993). The Clinical Dementia Rating (CDR): current version and scoring rules. Neurology.

[19] Morris JC, Heyman A, Mohs RC, Hughes JP, van Belle G, Fillenbaum G (1989). The Consortium to Establish a Registry for Alzheimer’s Disease (CERAD). Part I. Clinical and neuropsychological assessment of Alzheimer’s disease. Neurology..

[20] Lee JH, Lee KU, Lee DY, Kim KW, Jhoo JH, Kim JH (2002). Development of the Korean version of the Consortium to Establish a Registry for Alzheimer’s Disease Assessment Packet (CERAD-K): clinical and neuropsychological assessment batteries. J Gerontol B Psychol Sci Soc Sci.

[21] Lee DY, Lee KU, Lee JH, Kim KW, Jhoo JH, Kim SY (2004). A normative study of the CERAD neuropsychological assessment battery in the Korean elderly. J Int Neuropsychol Soc.

[22] Yesavage JA, Brink TL, Rose TL, Lum O, Huang V, Adey M (1982). Development and validation of a geriatric depression screening scale: a preliminary report. J Psychiatr Res.

[23] Kim JY, Park JH, Lee JJ, Huh Y, Lee SB, Han SK (2008). Standardization of the Korean version of the geriatric depression scale: reliability, validity, and factor structure. Psychiatry Investig.

[24] DeCarli C, Mungas D, Harvey D, Reed B, Weiner M, Chui H (2004). Memory impairment, but not cerebrovascular disease, predicts progression of MCI to dementia. Neurology.

[25] Friedenreich CM, Courneya KS, Bryant HE (1998). The lifetime total physical activity questionnaire: development and reliability. Med Sci Sports Exerc.

[26] Torres ER (2018). Validation of the Lifetime Total Physical Activity Questionnaire (LTPAQ) in midlife and older adults with a history of late-onset depression. Arch Psychiatr Nurs.

[27] Elsawy B, Higgins KE (2010). Physical activity guidelines for older adults. Am Fam Physician.

[28] Rovio S, Kareholt I, Helkala EL, Viitanen M, Winblad B, Tuomilehto J (2005). Leisure-time physical activity at midlife and the risk of dementia and Alzheimer’s disease. Lancet Neurol.

[29] Tolppanen AM, Solomon A, Kulmala J, Kareholt I, Ngandu T, Rusanen M (2015). Leisure-time physical activity from mid- to late life, body mass index, and risk of dementia. Alzheimers Dement.

[30] Choe MA, Kim J, Jeon M, Chae YR (2010). Evaluation of the Korean Version of Physical Activity Scale for the Elderly (K-PASE). Korean J Women Health Nurs.

[31] Washburn RA, Smith KW, Jette AM, Janney CA (1993). The Physical Activity Scale for the Elderly (PASE): development and evaluation. J Clin Epidemiol.

[32] Movement Disorder Society Task Force on Rating Scales for Parkinson’s D (2003). The Unified Parkinson’s Disease Rating Scale (UPDRS): status and recommendations. Mov Disord.

[33] Park K, Dankowicz H, Hsiao-Wecksler ET (2012). Characterization of spatiotemporally complex gait patterns using cross-correlation signatures. Gait Posture.

[34] Vellas B, Guigoz Y, Garry PJ, Nourhashemi F, Bennahum D, Lauque S (1999). The Mini Nutritional Assessment (MNA) and its use in grading the nutritional state of elderly patients. Nutrition.

[35] Rabin JS, Klein H, Kirn DR, Schultz AP, Yang HS, Hampton O (2019). Associations of Physical Activity and beta-Amyloid With Longitudinal Cognition and Neurodegeneration in Clinically Normal Older Adults. JAMA Neurol.

[36] Cotman CW, Berchtold NC (2002). Exercise: a behavioral intervention to enhance brain health and plasticity. Trends Neurosci.

[37] Turner PR, O’Connor K, Tate WP, Abraham WC (2003). Roles of amyloid precursor protein and its fragments in regulating neural activity, plasticity and memory. Prog Neurobiol.

[38] Lee TH, Jang MH, Shin MC, Lim BV, Kim YP, Kim H (2003). Dependence of rat hippocampal c-Fos expression on intensity and duration of exercise. Life Sci.

[39] Radak Z, Sasvari M, Nyakas C, Taylor AW, Ohno H, Nakamoto H (2000). Regular training modulates the accumulation of reactive carbonyl derivatives in mitochondrial and cytosolic fractions of rat skeletal muscle. Arch Biochem Biophys.

[40] Nunan J, Williamson NA, Hill AF, Sernee MF, Masters CL, Small DH (2003). Proteasome-mediated degradation of the C-terminus of the Alzheimer’s disease beta-amyloid protein precursor: effect of C-terminal truncation on production of beta-amyloid protein. J Neurosci Res.

[41] Lopez Salon M, Pasquini L, Besio Moreno M, Pasquini JM, Soto E (2003). Relationship between beta-amyloid degradation and the 26S proteasome in neural cells. Exp Neurol.

[42] Mendez Colmenares A, Voss MW, Fanning J, Salerno EA, Gothe NP, Thomas ML (2021). White matter plasticity in healthy older adults: the effects of aerobic exercise. Neuroimage.

[43] Palta P, Sharrett AR, Deal JA, Evenson KR, Gabriel KP, Folsom AR (2019). Leisure-time physical activity sustained since midlife and preservation of cognitive function: the Atherosclerosis Risk in Communities Study. Alzheimers Dement.

[44] Erickson KI, Hillman C, Stillman CM, Ballard RM, Bloodgood B, Conroy DE (2019). Physical activity, cognition, and brain outcomes: a review of the 2018 physical activity guidelines. Med Sci Sports Exerc.

[45] Tomata Y, Zhang S, Sugiyama K, Kaiho Y, Sugawara Y, Tsuji I (2017). Changes in time spent walking and the risk of incident dementia in older Japanese people: the Ohsaki Cohort 2006 Study. Age Ageing.

[46] Tomata Y, Zhang S, Sugawara Y, Tsuji I (2019). Impact of time spent walking on incident dementia in elderly Japanese. Int J Geriatr Psychiatry.

[47] Swain DP (2005). Moderate or vigorous intensity exercise: which is better for improving aerobic fitness?. Prev Cardiol.

[48] Morris JN, Chave SP, Adam C, Sirey C, Epstein L, Sheehan DJ (1973). Vigorous exercise in leisure-time and the incidence of coronary heart-disease. Lancet.

[49] Fassier P, Kang JH, Lee IM, Grodstein F, Vercambre MN (2022). Vigorous physical activity and cognitive trajectory later in life: prospective association and interaction by apolipoprotein E e4 in the Nurses’ Health Study. J Gerontol A Biol Sci Med Sci.

[50] Nakagawa T, Koan I, Chen C, Matsubara T, Hagiwara K, Lei H (2020). Regular moderate- to vigorous-intensity physical activity rather than walking is associated with enhanced cognitive functions and mental health in young adults. Int J Environ Res Public Health.

[51] Iso-Markku P, Waller K, Vuoksimaa E, Heikkila K, Rinne J, Kaprio J (2016). Midlife physical activity and cognition later in life: a prospective twin study. J Alzheimers Dis.

[52] Leyhe T, Muller S, Milian M, Eschweiler GW, Saur R (2009). Impairment of episodic and semantic autobiographical memory in patients with mild cognitive impairment and early Alzheimer’s disease. Neuropsychologia.

