# Supplementary material for: High-intensity walking in midlife is associated with improved memory in physically capable older adults
Source: Alzheimers Res Ther. 2023 Aug 29;15:143. doi: 10.1186/s13195-023-01293-8 (PMC10463890; doi:10.1186/s13195-023-01293-8)
Supplement: Supplementary file 1 — Additional file 1. [file 13195_2023_1293_MOESM1_ESM.docx]

| **Table S1.** Results of multiple linear regression analyses of the associations between number of years of walking practice and cognition (n = 188) | | | | | | | | | | | | | |  |
| --- | --- | --- | --- | --- | --- | --- | --- | --- | --- | --- | --- | --- | --- | --- |
|  |  |  | Total score | | | | Memory score | | | | Non-memory score | | | |
|  |  |  | B | SE | $\beta$ | *p* | B | SE | $\beta$ | *p* | B | SE | $\beta$ | *p* |
| Overall walking (n = 125) | | | | | | | | | | | | | |  |
| **Model 1** ^a^ |  |  |  | | |  |  | | |  |  | | |  |
| Number of years of walking practice |  |  | 0.074 | 0.090 | 0.062 | 0.411 | 0.045 | 0.053 | 0.063 | 0.397 | 0.009 | 0.040 | 0.018 | 0.815 |
| None |  |  | Reference | | |  | Reference | | |  | Reference | | |  |
| **Model 2** ^b^ |  |  |  | | |  |  | | |  |  | | |  |
| Number of years of walking practice |  |  | 0.037 | 0.079 | 0.031 | 0.641 | 0.032 | 0.047 | 0.045 | 0.496 | -0.014 | 0.036 | -0.027 | 0.694 |
| None |  |  | Reference | | |  | Reference | | |  | Reference | | |  |
| Midlife-initiated walking (n = 103) | | | | | | | | | | | | | |  |
| **Model 1** ^a^ |  |  |  | | |  |  | | |  |  | | |  |
| Number of years of walking practice |  |  | 0.091 | 0.099 | 0.076 | 0.360 | 0.048 | 0.057 | 0.070 | 0.400 | 0.023 | 0.043 | 0.044 | 0.599 |
| None |  |  | Reference | | |  | Reference | | |  | Reference | | |  |
| **Model 2** ^b^ |  |  |  | | |  |  | | |  |  | | |  |
| Number of years of walking practice |  |  | 0.063 | 0.085 | 0.053 | 0.460 | 0.045 | 0.051 | 0.066 | 0.375 | -0.002 | 0.038 | -0.003 | 0.965 |
| None |  |  | Reference | | |  | Reference | | |  | Reference | | |  |
| Late life-initiated walking (n = 22) | | | | | | | | | | | | | |  |
| **Model 1** ^a^ |  |  |  | | |  |  | | |  |  | | |  |
| Number of years of walking practice |  |  | 0.306 | 0.396 | 0.088 | 0.442 | 0.263 | 0.255 | 0.114 | 0.306 | -0.081 | 0.185 | -0.051 | 0.662 |
| None |  |  | Reference | | |  | Reference | | |  | Reference | | |  |
| **Model 2** ^b^ |  |  |  | | |  |  | | |  |  | | |  |
| Number of years of walking practice |  |  | 0.131 | 0.388 | 0.038 | 0.736 | 0.157 | 0.257 | 0.068 | 0.543 | -0.174 | 0.181 | -0.110 | 0.340 |
| None |  |  | Reference | | |  | Reference | | |  | Reference | | |  |
| APOE4 apolipoprotein ε4, GDS geriatric depression scale, VRS vascular risk score, BMI body mass index.  For these analyses, we used the no walking (n = 63) as the reference.  ^a^ Adjusted for age, sex, and APOE4.  ^b^ Adjusted for covariates in Model 1 plus, education, clinical diagnosis, GDS, annual income, alcohol intake, smoking, VRS, BMI, dietary pattern including food types (such as protein and fruit or vegetables), serum nutritional markers (such as hemoglobin, albumin, glucose, and HDL-/LDL-cholesterol) and overall physical activity score. | | | | | | | | | | | | | |  |

| **Table S2.** Results of multiple linear regression analyses including number of years of walking practice$\times$one covariate interaction term, predicting cognition (n = 188). | | | | | | | | | | | | |
| --- | --- | --- | --- | --- | --- | --- | --- | --- | --- | --- | --- | --- |
|  | Total score | | | | Memory score | | | | Non-memory score | | | |
|  | B | SE | $\beta$ | *p* | B | SE | $\beta$ | *p* | B | SE | $\beta$ | *p* |
| Number of years of walking practice | 1.548 | 0.894 | 1.291 | 0.086 | 1.106 | 0.627 | 1.559 | 0.080 | 0.718 | 0.465 | 1.396 | 0.125 |
| Age | -0.244 | 0.224 | -0.080 | 0.279 | -0.153 | 0.158 | -0.085 | 0.333 | -0.045 | 0.117 | -0.034 | 0.700 |
| Number of years of walking practice$\times$ Age | -0.021 | 0.012 | -0.1296 | 0.086 | -0.015 | 0.009 | -1.527 | 0.088 | -0.010 | 0.006 | -1.447 | 0.114 |
| APOE4 apolipoprotein ε4, GDS geriatric depression scale, VRS vascular risk score, BMI body mass index, PA physical activity. | | | | | | | | | | | | |
